# A Meta-Analysis Examining the Effect of Perioperative Biologic Disease-Modifying Anti-Rheumatic Medications on Postoperative Wound Complications in Various Orthopedic Surgeries

**DOI:** 10.3390/jcm13185531

**Published:** 2024-09-18

**Authors:** Mohamed S. Imam, Shahad A. Alshahrani, Rahaf Mubarak S. Alotaibi, Khalid Nassir Almurayeh, Naif Ayidh Alshabab, Nora Khaled Almutairi, Hanin Alomar, Leen Alomair, Marina E. Boules, Mohamed E. Abdelrahim, Mohammed A. Amin

**Affiliations:** 1 Department of Clinical Pharmacy, College of Pharmacy, Shaqra University, Shaqra 11961, Saudi Arabia; 2 Department of Clinical Pharmacy, National Cancer Institute, Cairo University, Fom El Khalig Square, Kasr Al-Aini Street, Cairo 11796, Egypt; 3 College of Pharmacy, Taif University, Taif 21944, Saudi Arabia; 4 Al-Jazea Medical Company, Riyadh 11474, Saudi Arabia; 5 College of Pharmacy, King Khalid University, Abha 62529, Saudi Arabia; 6 College of Pharmacy, Princess Nourah Bint Abdulrahman University, Riyadh 11671, Saudi Arabia; 7 Clinical Pharmacy Department, Faculty of Pharmacy, Beni-Suef University, Beni Suef 65211, Egypt

**Keywords:** orthopedic surgery, delayed wound healing, biologic disease-modifying anti-rheumatic drug, post-operative infection, disease flare

## Abstract

This meta-analysis was designed to compare the risk of postoperative wound complications in various orthopedic surgeries (OSs) affected by the perioperative use of biologic disease-modifying anti-rheumatic drugs (bDMARDs). The odds ratio (OR) and mean difference (MD), with 95% confidence intervals (CIs), were calculated using dichotomous or continuous random or fixed-effect models, based on the meta-analysis data. This study incorporated 14 investigations conducted between 2005 and 2023, encompassing a total population of 19,021 individuals undergoing diverse OSs. Participants who continued their bDMARDs exhibited a substantially higher incidence of postoperative surgical site infections (OR, 1.39; 95% CI, 1.12–1.72, *p* = 0.002) compared to those who withheld bDMARDs. However, the study did not find any statistically significant difference between the continuation or withholding of bDMARDs regarding delayed wound healing (OR, 2.02; 95% CI, 1.00–4.06, *p* = 0.05) or disease flares (OR, 0.59; 95% CI, 0.28–1.25, *p* = 0.17). The results show that patients who continued their bDMARDs had a notably higher incidence of postoperative surgical site infections. However, no significant differences were observed in delayed wound healing or disease flares when compared to those who withheld bDMARDs. It is important to acknowledge the limitations of this analysis, such as the relatively small number of participants and the limited number of studies available for certain comparisons, which may impact the validity of the findings.

## 1. Introduction

Rheumatoid arthritis affects approximately 0.5% to 1% of the global population, while psoriatic arthritis affects around 0.16% [1]. These inflammatory rheumatic illnesses are common systemic autoimmune disorders [1], with rheumatoid arthritis impacting over 10 million individuals worldwide, often leading to a substantial decline in their quality of life. Disease-modifying anti-rheumatic drugs (DMARDs) have been proven to be effective and well-established treatments for these rheumatic conditions. DMARDs are categorized into two main types: biologic DMARDs (bDMARDs), such as tumor necrosis factor inhibitors (TNFis), and conventional synthetic DMARDs, such as methotrexate. The use of bDMARDs has been on the rise, as evidenced by the fact that up to 44% of individuals with rheumatoid arthritis who undergo joint arthroplasty are prescribed bDMARDs prior to the procedure [2].

Orthopedic operations are more common in patients with inflammatory arthropathies [3], as these conditions are characterized by structural damage to both joints and tissues [4].

bDMARDs slow the progression of the disease both clinically and radiographically, but they also increase the risk of infection. In a meta-analysis, Ito et al. [5] found that patients on bDMARDs had a slightly higher relative risk of surgical site infections (SSIs) after orthopedic surgery (OS) but not a higher risk of delayed wound healing. Periprosthetic joint infections are generally 50% to 80% more common in individuals with rheumatoid arthritis than in those with osteoarthritis [6]. The optimal approach for the perioperative management of bDMARDs is still under investigation, given the serious consequences and challenges associated with infection management. Current guidelines recommend withholding bDMARDs before surgery [7]. While this approach significantly impacts the patient’s overall well-being and quality of life, it also carries the risk of poor disease control (i.e., “flares”). Collaboration between surgeons, rheumatologists, and patients is essential to navigate the difficult trade-off between the risk of infection while on medication and the likelihood of experiencing a flare and its associated complications. Patients with rheumatoid arthritis frequently experience flares, which can be triggered by discontinuing medication [8]. It has been shown that more than 60% of rheumatoid arthritis patients undergoing arthroplasty may experience a flare-up [8]. Currently, there is conflicting information regarding when and if bDMARDs should be stopped and resumed in the time leading up to surgery. While some evidence suggests that bDMARDs can be safely continued, other studies have found an increased susceptibility to infection with prolonged use [9]. Patients who discontinued bDMARDs after surgery experienced a reduced occurrence of infections [10]. However, this conclusion was based on data from only three trials, and additional studies have since been published [11]. Previous research has demonstrated that individuals using bDMARDs are more susceptible to infection than those not receiving them. The primary aim of the present meta-analysis was to assess and evaluate the likelihood of postoperative surgical site infections (SSIs), delayed wound healing, and disease flares among patients who either maintained or ceased the use of bDMARDs during the perioperative phase.

## 2. Methods

### 2.1. Design of the Examination

**Figure 1:**
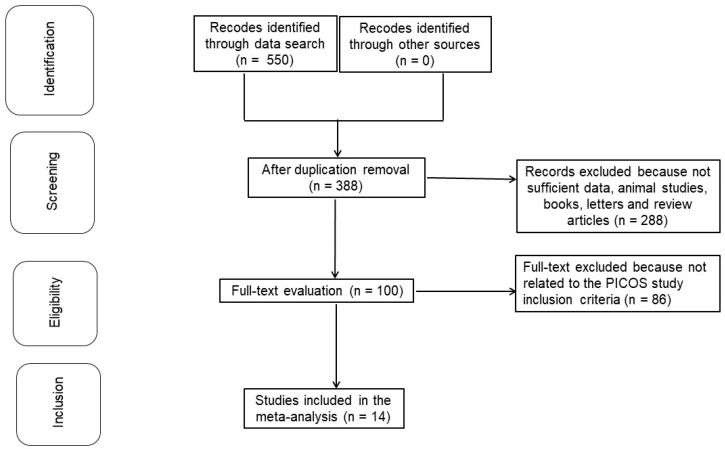
Schematic diagram of the examination procedure.

The meta-analysis was conducted using a predetermined methodology and incorporated into the epidemiological framework. Data collection and analysis were performed using multiple databases, including OVID, PubMed, the Cochrane Library, Embase, and Google Scholar. These datasets provided the foundation for comparisons and risk assessments of postoperative wound complications in various orthopedic surgeries exposed to perioperative bDMARDs [12]. Data pooling bDMARD has been observed to produce various clinical outcomes across different operating systems. The primary outcome considered in the inclusion criteria for these studies was postoperative surgical site infection (SSI). Language constraints were not applied during the selection of studies or the screening of potential participants. Additionally, no restrictions were placed on the number of participants recruited for the investigations. Reviews, editorials, and letters were excluded from our synthesis due to the absence of objective data. The detailed identification process for the comprehensive examination is illustrated in Figure 1.

### 2.2. Eligibility of Included Studies

An investigation is currently underway to determine whether bDMARDs have a beneficial or detrimental impact on the clinical outcomes of various orthopedic surgeries (OSs). The sensitivity analysis included only studies that assessed the effect of treatments on the frequency of OSs. Both sensitivity and subgroup analyses were performed by comparing the interventional groups across different subtypes.

### 2.3. Inclusion Criteria

For a study to be included in the meta-analysis, it had to compare the outcomes of continuing versus discontinuing bDMARDs in terms of reducing the incidence of surgical site infections (SSIs) following various types of surgery. To enable statistical analysis, the outcome measures needed to be clearly expressed in the study’s results.

### 2.4. Exclusion Criteria

Exclusion criteria included non-comparative study designs. Additionally, letters, books, reviews, and book chapters were not included in the current assessment.

### 2.5. Identification of Studies

A search protocol was developed and defined using the PICOS principle [13], which is outlined as follows: P (population): individuals undergoing various orthopedic surgeries (OSs); I (Intervention/Exposure): use of bDMARDs; C (Comparison): continuation versus discontinuation of bDMARDs; O (Outcome): incidence of postoperative surgical site infections (SSIs); S (Study Design): the assessment was not restricted to any specific study design [13].

A comprehensive search was conducted across relevant databases up until September 2023, using the keywords and associated terms listed in Table 1. All papers included in the reference management program underwent thorough reviews, including abstracts, titles, and full texts, to ensure a connection between the treatment type and clinical outcomes. Additionally, two authors independently assessed the articles to identify relevant studies [14].

### 2.6. Screening of Studies

The data were streamlined by considering various factors, including the study design, standardized presentation of individual characteristics, first author’s surname, date and year of the study, country of administration, participant gender, population type, total number of participants, qualitative and quantitative evaluation methods, demographic information, and clinical and treatment characteristics [15]. Two independent authors assessed the potential presence of bias in each study, as well as the quality of the methodologies used in the selected studies for further analysis. The methodologies employed in each assessment were objectively reviewed by both authors [16]. 

In the current meta-analysis, the researchers employed dichotomous or continuous random- or fixed-effect models to calculate the odds ratio (OR) and mean difference (MD) with a 95% confidence interval (CI). The I2 index, expressed as a percentage ranging from 0 to 100 [15], was used to assess heterogeneity. Higher I2 values indicate increased heterogeneity, while lower values suggest a lack of heterogeneity. A random-effects model was chosen when I2 exceeded 50%, and a fixed-effects model was applied when I2 was below 50% [17]. The outcomes of the initial inquiry were categorized within the framework of the subcategory analysis, as previously described. Begg’s and Egger’s tests were used for quantitative analysis to assess publication bias, which was considered significant if the *p*-value was greater than 0.05. *p*-values were calculated using a two-tailed approach. Jamovi 2.3 was utilized for graph creation and statistical analysis.

## 3. Results

A total of fourteen investigations published between 2006 and 2023 were deemed suitable for inclusion in the meta-analysis after reviewing 550 pertinent papers [18,19,20,21,22,23,24,25,26,27,28,29,30,31].

Table 2 summarizes the findings of these investigations. Among the 19,021 individuals who underwent various orthopedic surgeries, 4830 continued their bDMARDs, while 14,191 withheld their bDMARDs.

**Figure 2:**
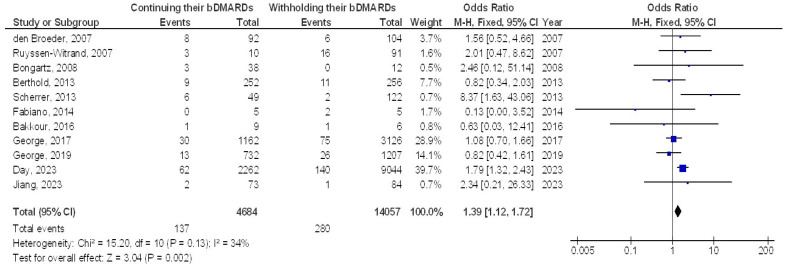
The effect’s forest plot of the continuing their bDMARDs compared to withholding their bDMARDs on postoperative surgical site infection in orthopedic surgery subjects [20,21,22,23,24,25,26,27,28,29,31].

Continuing bDMARDs was associated with a significantly higher incidence of postoperative surgical site infections (SSIs) (OR, 1.39; 95% CI, 1.12–1.72, *p* = 0.002) with low heterogeneity (I^2^ = 34%) compared to withholding bDMARDs in orthopedic surgery patients, as illustrated in Figure 2.

**Figure 3:**
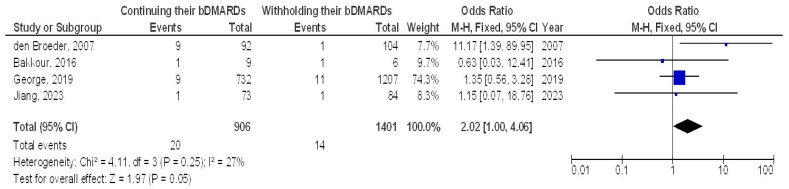
The effect’s forest plot of continuing their bDMARDs compared to withholding their bDMARDs on delayed wound healing in orthopedic surgery subjects [18,20,28,31].

**Figure 4:**
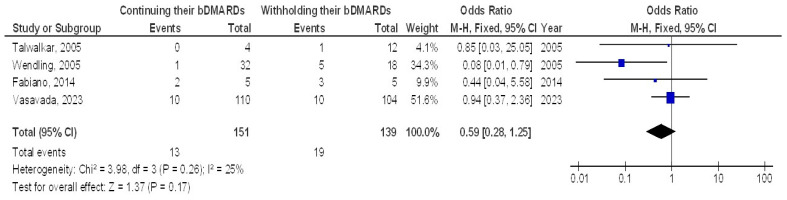
The effect’s forest plot of continuing their bDMARDs compared to withholding their bDMARDs on disease flares in orthopedic surgery subjects [18,19,25,30].

However, no significant difference was found between continuing and withholding bDMARDs in terms of delayed wound healing (OR, 2.02; 95% CI, 1.00–4.06, *p* = 0.05) with low heterogeneity (I^2^ = 27%) or in the incidence of disease flares (OR, 0.59; 95% CI, 0.28–1.25, *p* = 0.17) with low heterogeneity (I^2^ = 25%), as illustrated in Figure 3 and Figure 4.

**Figure 5:**
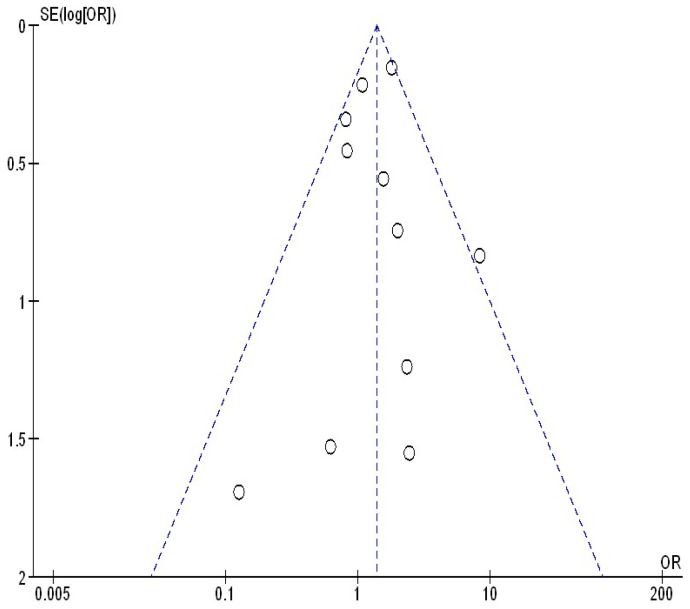
The funnel plot of continuing their bDMARDs compared to withholding their bDMARDs on postoperative surgical site infection in orthopedic surgery subjects.

**Figure 6:**
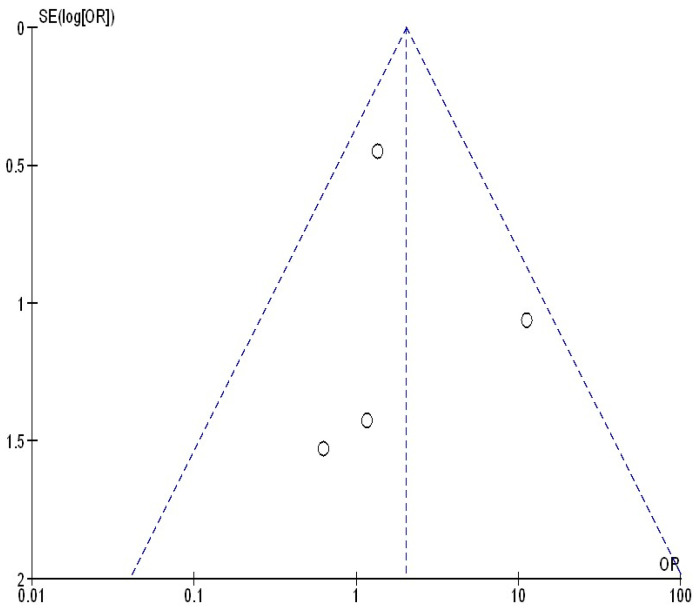
The funnel plot of continuing their bDMARDs compared to withholding their bDMARDs on delayed wound healing in orthopedic surgery subjects.

**Figure 7:**
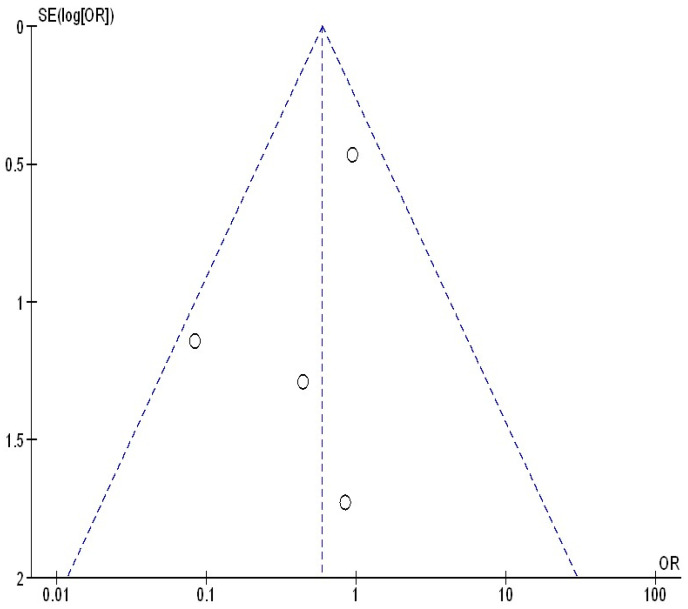
The funnel plot of continuing their bDMARDs compared to withholding their bDMARDs on disease flares in orthopedic surgery subjects.

**Figure 8:**
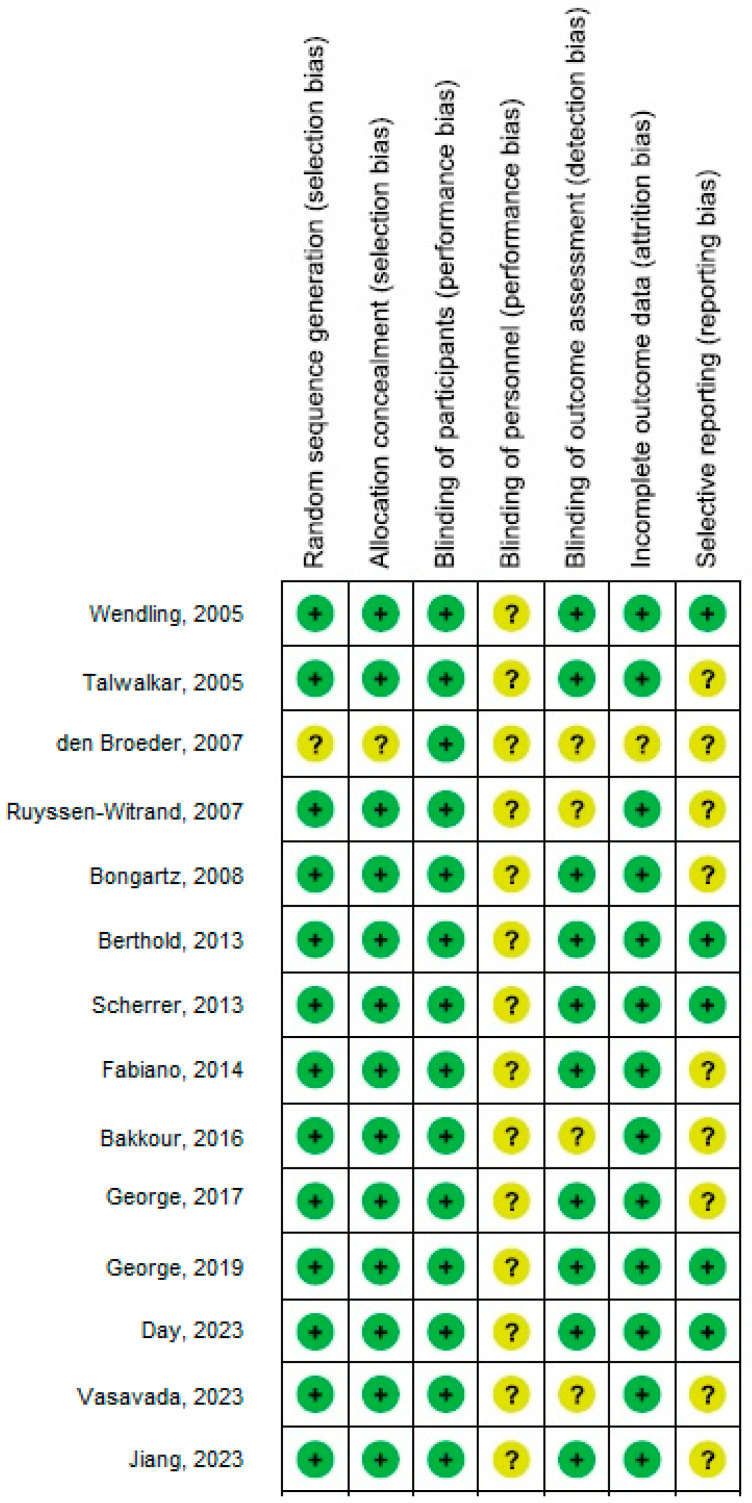
Risk of bias summary for each included study [18,19,20,21,22,23,24,25,26,27,28,29,30,31].

Figure 5, Figure 6 and Figure 7 illustrate that there was no evidence of examination bias in the quantitative Egger regression test or the visual interpretation of the effect’s forest plot (*p* = 0.87). However, the findings indicated that most relevant studies exhibited reporting bias and were of low practical quality, as shown in Figure 8.

## 4. Discussion

The current meta-analysis includes 14 studies conducted between 2005 and 2023, encompassing a total of 19,021 individuals who underwent various orthopedic surgeries. Of these, 4830 continued their bDMARDs, while 14,191 withheld them. The sample sizes of the studies ranged from 10 to 11,306 participants [18,19,20,21,22,23,24,25,26,27,28,29,30,31]. The analyzed data revealed a significant disparity in the risk of postoperative surgical site infections (SSIs) between patients who continued their bDMARDs and those who discontinued them. However, no statistically significant differences were observed between the continuation and cessation of bDMARDs concerning delayed wound healing and disease flares. It is important to note the limited sample size in some studies—specifically, 5 out of the 14 studies had fewer than 100 subjects—as well as the small number of studies selected for certain comparisons. Therefore, the significance of these findings should be interpreted with caution.

For patients with inflammatory diseases, the goal of prescribing DMARDs—both conventional and biological—is to achieve either sustained low disease activity or disease remission. However, because bDMARDs alter the host immune response, individuals who are already at a higher risk of postoperative surgical site infections (SSIs) compared to the general population may face an even greater risk. Previous meta-analyses have examined the hazards of SSIs in patients taking bDMARDs for OSs [32,33,34,35,36]. Goodman et al. conducted a study on patients with rheumatoid arthritis who underwent elective orthopedic surgery, comparing those who had not recently been exposed to TNFis with those who had been exposed to TNFis within three months of surgery [3]. They found that the latter group had a higher risk of developing SSIs. However, because patients requiring TNFi medication probably have more severe disease, which is another known risk factor for infection [37], the group in that study is not directly comparable to the population in the current analysis. In another study, Ito et al. [5] evaluated the outcomes of rheumatoid arthritis patients undergoing elective orthopedic surgery. They found that patients on bDMARDs had a higher relative risk of SSIs, but there was no increase in the rate of delayed wound healing in this patient population. It is well known that discontinuing bDMARDs can increase the likelihood of a flare-up characterized by fatigue and pain.

Flare-ups of the disease can make recovery from arthroplasty surgery more challenging for patients. Additionally, patients experiencing a flare-up often require corticosteroids, which further increases the risk of infection [38]. Therefore, if bDMARDs are continued throughout the perioperative phase, it is crucial to balance the risk of infection against the risk of flares. In response to these concerns, the British Society for Rheumatology, the American College of Rheumatology, and the American Association of Hip and Knee Surgeons issued guidelines in 2017 and 2019 recommending that biologic drugs be withheld during the perioperative phase [7,39]. However, the American Association of Hip and Knee Surgeons’ guidelines emphasize that the available evidence is of low quality, and no definitive conclusions—such as those from randomized controlled trials—exist to determine when or whether to discontinue biologic medication-assisted therapy. Part of the evidence for these guidelines was derived from randomized controlled trials that did not involve surgery, as well as feedback from patients who were convinced that the risk of infection outweighed the risk of a flare-up. Following the publication of these guidelines, George et al. examined a large cohort of patients undergoing arthroplasty to assess the likelihood of infection while taking abatacept or infliximab. They found no association between discontinuing these drugs before surgery and a reduced risk of infection [27,28]. Clay et al. also assessed the frequencies of SSIs, flare-ups, and postoperative complications in patients with rheumatoid arthritis [10].

In the only other systematic review to date, it was found that patients who continued their TNFis had a higher risk of SSIs, while those who withheld their TNFis had a significantly higher risk of disease flares. Bakkour et al. evaluated 42 patients with psoriasis and psoriatic arthritis undergoing various surgical procedures [26] and found that patients who stopped taking bDMARDs had a significantly higher risk of experiencing a flare-up related to their disease. More recently, Goodman et al. evaluated the clinical features of 120 patients with rheumatoid arthritis at 0 to two weeks preoperatively and six weeks postoperatively. Among them, 61 patients (51%) had been using bDMARDs preoperatively and were instructed to stop taking them but to continue methotrexate and glucocorticoids postoperatively. Sixty-three percent of the entire cohort reported disease flares. Interestingly, patients who experienced flare-ups did not differ significantly in their use of methotrexate or glucocorticoids compared to those who did not experience flares. Notably, baseline disease activity was higher in patients at the highest risk of flare. The authors concluded that while a higher percentage of patients not using bDMARDs experienced disease flares, discontinuing the treatment was not identified as an independent risk factor for flares. However, it is important to note that the study was not specifically designed to examine the continuation or cessation of bDMARDs.

Part of the evidence for these guidelines came from randomized controlled trials unrelated to surgery, along with feedback from a group of patients who believed the risk of infection outweighed the chance of a flare-up. This is further emphasized in meta-analysis and predictive clinical assessment [40,41].

The meta-analysis faced several constraints: the potential for selection bias and heterogeneity, which could be due to the exclusion of certain studies that did not meet the inclusion criteria. Additionally, data were needed to assess whether variables such as age, gender, and ethnicity affected the outcomes. The primary objective of the meta-analysis was to determine the likelihood of postoperative wound complications in various orthopedic surgeries affected by preoperative bDMARD use.

The use of erroneous or inadequate data from previous investigations likely exacerbated bias. Discrimination was likely affected by factors such as age, gender, ethnicity, and nutritional status. Incomplete data and unreported investigations can inadvertently alter values, leading to skewed results. Also, the lack of information on the timing of bDMARD discontinuation and the duration of bDMARD use before surgery were some additional limitations of our meta-analysis.

## 5. Conclusions

The data analysis revealed that patients who continued their bDMARDs experienced a significantly higher incidence of postoperative surgical site infections (SSIs) compared to those who withheld their bDMARDs in orthopedic surgery cases. However, no significant differences were observed between continuing or withholding bDMARDs regarding delayed wound healing and illness flares. It is important to note the limited sample size in certain studies—specifically, 5 out of the 14 studies had fewer than 100 subjects—and the small number of studies included for certain comparisons. These factors should be considered when evaluating the significance of the findings.

## Figures and Tables

**Table 1:** Database search strategy for the inclusion of examinations.

Database	Search Strategy
Google Scholar	#1 “orthopedic surgery” OR “delayed wound healing” #2 “biologic disease-modifying anti-rheumatic drug” OR “post-operative infection” OR “ “disease flare” #3 #1 AND #2
Embase	#1 ‘orthopedic surgery’/exp OR ‘delayed wound healing’/exp OR ‘disease flare’ #2 ‘biologic disease-modifying anti-rheumatic drug’/exp OR ‘post-operative infection’/ #3 #1 AND #2
Cochrane library	#1 (orthopedic surgery):ti,ab,kw (delayed wound healing):ti,ab,kw (disease flare):ti,ab,kw (Word variations have been searched) #2 (biologic disease-modifying anti-rheumatic drug):ti,ab,kw OR (post-operative infection):ti,ab,kw (Word variations have been searched) #3 #1 AND #2
Pubmed	#1 “orthopedic surgery” [MeSH] OR “delayed wound healing” [MeSH] OR “disease flare” [All Fields] #2 “biologic disease-modifying anti-rheumatic drug” [MeSH Terms] OR “post-operative infection” [All Fields] #3 #1 AND #2
OVID	#1 “orthopedic surgery” [All Fields] OR “delayed wound healing” [All Fields] OR “disease flare” [All Fields] #2 “biologic disease-modifying anti-rheumatic drug” [All fields] OR “post-operative infection” [All Fields] #3 #1 AND #2

**Table 2:** Characteristics of studies.

Study	Country	Total	Continuing Their bDMARDs	Withholding Their bDMARDs			
Wendling, 2005 [18]	France	50	32	18	Diagnosis	Procedures	Biologic(s) Included
Talwalkar, 2005 [19]	Multi-centered	16	4	12	rheumatoid arthritis, psoriatic arthritis	Hip and knee arthroplasty (primary/revision)	Abatacept
den Broeder, 2007 [20]	The Netherlands	196	92	104	rheumatoid arthritis	Various orthopaedic	Infliximab
Ruyssen-Witrand, 2007 [21]	France	101	10	91	rheumatoid arthritis, ankylosing spondylitis	Various orthopaedic	Etanercept, adalimumab, infliximab
Bongartz, 2008 [22]	USA	50	38	12	rheumatoid arthritis	Hip and knee arthroplasty (primary/revision)	Etanercept, adalimumab, and infliximab, rituximab, abatacept
Berthold, 2013 [23]	Sweden	508	252	256	rheumatoid arthritis	Various orthopaedic	Abatacept
Scherrer, 2013 [24]	Switzerland	171	49	122	rheumatoid arthritis, ankylosing spondylitis, juvenile arthritis, psoriatic arthritis	Hip and knee arthroplasty	Infliximab
Fabiano, 2014 [25]	Italy	10	5	5	psoriasis	Various orthopaedic	Abatacept
Bakkour, 2016 [26]	UK	15	9	6	rheumatoid arthritis	Various, including skin, cardiovascular, gastrointestinal, ear, nose, and throat, maxillofacialas well as orthopaedics	Infliximab, adalimumab, ustekinumab, etanercept
George, 2017 [27]	USA	4288	1162	3126	rheumatoid arthritis	Hip and knee arthroplasty	Abatacept
George, 2019 [28]	USA	1939	732	1207	rheumatoid arthritis	Various orthopaedic	Infliximab
Day, 2023 [29]	USA	11,306	2262	9044	rheumatoid arthritis	Hip and knee arthroplasty (primary/revision)	Abatacept
Vasavada, 2023 [30]	USA	214	110	104	rheumatoid arthritis	Hip and knee arthroplasty	Infliximab, etanercept, adalimumab
Jiang, 2023 [31]	China	157	73	84	rheumatoid arthritis	Hip and knee arthroplasty (primary/revision)	Abatacept
	**Total**	**19,021**	**4830**	**14,191**			
